# Effect of resuscitative endovascular balloon occlusion of the aorta in hemodynamically unstable patients with multiple severe torso trauma: a retrospective study

**DOI:** 10.1186/s13017-018-0210-5

**Published:** 2018-10-25

**Authors:** Hiroyuki Otsuka, Toshiki Sato, Keiji Sakurai, Hiromichi Aoki, Takeshi Yamagiwa, Shinichi Iizuka, Sadaki Inokuchi

**Affiliations:** Department of Emergency and Critical Care Medicine, Tokai University School of Medicine, 143 Shimokasuya, Isehara city, Kanagawa Prefecture 259-1193 Japan

**Keywords:** Resuscitative endovascular balloon occlusion of the aorta, Multiple lethal trauma, Resuscitation, Trauma management

## Abstract

**Background:**

Although resuscitative endovascular balloon occlusion of the aorta (REBOA) may be effective in trauma management, its effect in patients with severe multiple torso trauma remains unclear.

**Methods:**

We performed a retrospective study to evaluate trauma management with REBOA in hemodynamically unstable patients with severe multiple trauma. Of 5899 severe trauma patients admitted to our hospital between January 2011 and January 2018, we selected 107 patients with severe torso trauma (Injury Severity Score > 16) who displayed persistent hypotension [≥ 2 systolic blood pressure (SBP) values ≤ 90 mmHg] regardless of primary resuscitation. Patients were divided into two groups: trauma management with REBOA (*n* = 15) and without REBOA (*n* = 92). The primary endpoint was the effectiveness of trauma management with REBOA with respect to in-hospital mortality. Secondary endpoints included time from arrival to the start of hemostasis. Multivariable logistic regression analysis, adjusted for clinically important variables, was performed to evaluate clinical outcomes.

**Results:**

Trauma management with REBOA was significantly associated with decreased mortality (adjusted odds ratio of survival, 7.430; 95% confidence interval, 1.081–51.062; *p* = 0.041). The median time (interquartile range) from admission to initiation of hemostasis was not significantly different between the two groups [with REBOA 53.0 (40.0–80.3) min vs. without REBOA 57.0 (35.0–100.0) min ]. The time from arrival to the start of balloon occlusion was 55.7 ± 34.2 min. SBP before insertion of REBOA was 48.2 ± 10.5 mmHg. Total balloon occlusion time was 32.5 ± 18.2 min.

**Conclusions:**

The use of REBOA without a delay in initiating resuscitative hemostasis may improve the outcomes in patients with multiple severe torso trauma. However, optimal use may be essential for success.

## Background

Recently, new concepts and technologies such as damage-control strategies, whole-body computed tomography (CT), endovascular treatment, and hybrid operating rooms have been developed for the treatment of trauma patients [1–7]. Similarly, resuscitative endovascular balloon occlusion of the aorta (REBOA) has been widely used in the management of hemorrhagic shock [8]. Its effectiveness for trauma patients has been evaluated in many large-scale studies [9–13]; however, the evidence base is weak and clear indications are lacking. Furthermore, although the time and place of balloon insertion, zone of balloon inflation, and inflation cutoff time are very important, they are heterogeneous factors [11, 14–18]. In addition, while it has been conceivable that REBOA may be effective in patients with severe trauma when integrated with surgery or interventional radiology (IVR) without delay [9], it remains challenging to successfully perform REBOA in patients with severe multiple torso traumas.

The aim of this study was to evaluate our trauma management with REBOA in hemodynamically unstable patients with multiple severe trauma.

## Methods

### Study design and selection criteria

**Fig. 1:**
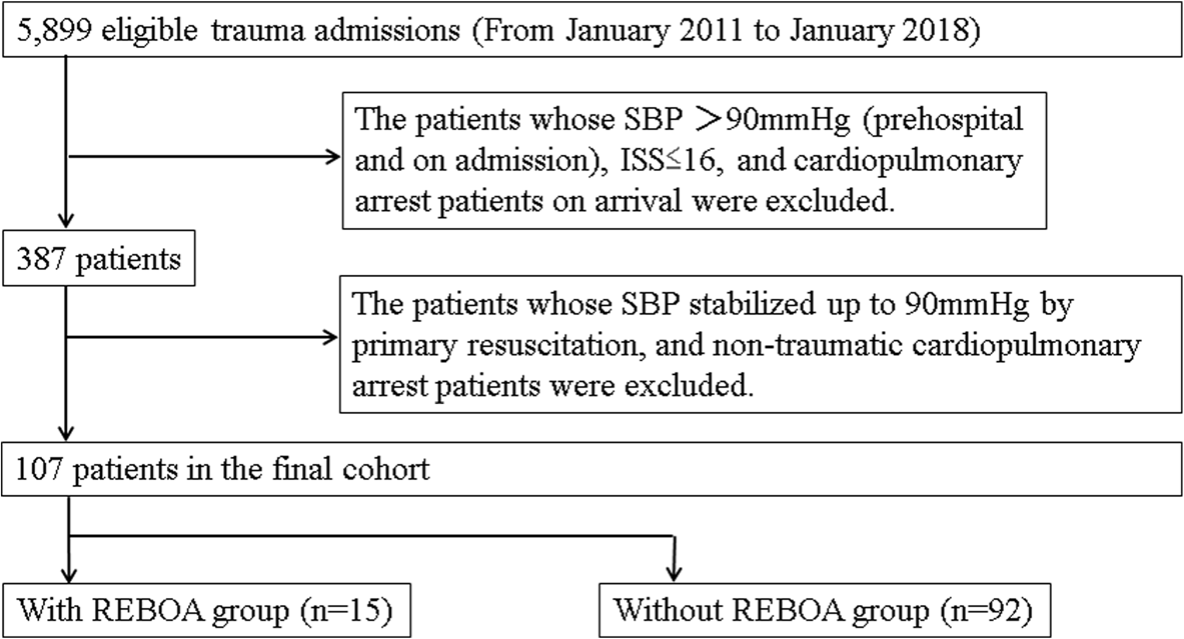
Flow diagram of patient inclusion in the study

A total of 5899 severe trauma patients were admitted to our hospital between January 2011 and January 2018. Among them, we selected 107 patients with severe torso trauma [Injury Severity Score (ISS) > 16] who displayed persistent hypotension [≥ 2 systolic blood pressure (SBP) values ≤ 90 mmHg] regardless of primary resuscitation (airway management, massive transfusion, and/or reversal of obstructive shock) without cardiopulmonary arrest on admission. The patients were divided into two groups: trauma management with REBOA or without REBOA (Fig. 1). The with REBOA group indicated REBOA used primarily, and not secondarily, after open aortic cross-clamping via resuscitative thoracotomy as part of the cardiopulmonary resuscitation procedure. We retrospectively evaluated the characteristics of the patients, hematological tests at the time of admission, severity of trauma, treatment-related characteristics, and outcomes. We defined the primary endpoint as the effectiveness of REBOA for in-hospital mortality. In addition, we set the following secondary endpoints to evaluate the effectiveness of REBOA: pre-hemostasis CT scan performance ratio, REBOA performance ratio before CT, ratio of patients who underwent hemostasis, total amount of red blood cells (RBCs) and fresh frozen plasma (FFP) transfused, ratio of patients who underwent surgery as primary hemostasis among those who underwent hemostasis, time course from arrival to the start of surgery/IVR, pre-hemostasis-administrated RBCs and FFP, and REBOA-related characteristics.

### REBOA procedure

The use of REBOA at our institution was started between 20 and 25 years ago. The decision to use REBOA and performing the procedure was at the discretion of the emergency physicians (EPs) or trauma surgeons. All EPs in our hospital have completed a 3-month course of radiology training (mainly vascular IVR). We have used REBOA in patients with severe multiple trauma as follows: the use of REBOA should never result in any delay in the initiation of resuscitative surgery/IVR; REBOA is used to prevent cardiac arrest and not to improve the shock state in patients with severe hemorrhagic shock. However, we do not make any guidelines for the use of REBOA. REBOA has been used under simply primary doctor judgment. In our center, most trauma patients who deteriorated into a state of severe shock have received an insertion of 4-French sheath into the common femoral artery, for the continuous measurement of arterial pressure or performing IVR irrespective of whether REBOA was used or not. Femoral access was obtained through anatomic landmarks using sonography without a surgical incision. REBOA was performed by inserting a 12-French or 7-French sheath set in the common femoral artery. Through the sheath, a balloon catheter was blindly inserted and initially inflated in the aortic zone I [19], located between the left subclavian artery and the celiac artery.

### Trauma management

Our institution is a tertiary referral hospital that includes a specialized department in trauma management and intensive care. Moreover, the CT scan, angiography suite (AS), and operating room (OR) are part of the emergency department (ED) in our hospital and available for use at all hours. All trauma surgeons in our hospital have been trained in emergency medicine and general surgery and are also trained in cardiovascular surgery and IVR. The decision to perform surgery or IVR was made at the discretion of EPs or the trauma surgeons in the ED.

### Data collection

The ED variables [Glasgow Coma Scale (GCS), respiratory rate (RR), SBP, body temperature (BT), pulse rate, pH, base excess, lactate value, D-dimer, and prothrombin time-international normalized ratio] were recorded as the initial set of vital signs and laboratory tests. Revised Trauma Score (RTS), ISS, and probability of survival calculated using the Trauma and Injury Severity Score (TRISS-Ps) were used for analyzing patient characteristics and severity.

### Statistical analysis

Statistical analyses were performed using SPSS software (Windows version 22.0; SPSS Inc., Chicago, IL, USA). For the primary endpoint, multivariable logistic regression analysis was performed to evaluate the effectiveness of REBOA before cardiopulmonary arrest after adjusting for age, RTS, ISS, pre-hemostasis-administrated RBCs, and the logarithm of time from admission to the start of hemostasis. For patients' baseline characteristics and secondary endpoints, categorical variables were compared using the *χ*^2^ test or Fisher’s exact test, and continuous variables were analyzed using the Student’s *t* test or the Mann–Whitney *U* test. The values are presented as either the mean ± standard deviation or the median (interquartile range [IQR] 25–75). Statistical significance was defined as a *p* value of less than 0.05 or was assessed using 95% confidence intervals (CI).

This study was approved by the institutional review board for clinical research, Tokai University (approval no.: 17R-344).

## Results

Fifteen patients were included in the with REBOA group, and 92 patients were included in the without REBOA group. Table 1 summarizes the patients’ baseline characteristics. There were no significant differences between the two groups.

**Table 1:** Characteristics and severity

	With REBOA (*n* = 15)	Without REBOA (*n* = 92)	*p*
Age, years	52.7 ± 19.8	52.1 ± 21.0	0.67
Male gender (%)	11 (73.3)	59 (64.1)	0.487
Mechanism of injury			
Motor vehicle accident	9	41	0.977
Fall from a height	5	34	
Stabbing	0	11	
Compression	1	4	
Gunshot	0	1	
Violence	0	1	
Vital signs on admission			
GCS total score	11.0 (3.0–14.0)	9.0 (4.25–14.0)	0.83
GCS < 9 (%)	6 (40.0)	45 (48.9)	0.522
RR, per min	24.0 (24.0–30.0)	24.0 (18.0–30.0)	0.643
SBP, mmHg	60.0 (52.0–90.0)	71.0 (56.5–84.5)	0.474
BT, Celsius	36.0 (35.0–36.8)	36.0 (35.4–36.7)	0.804
Pulse rate, beats per min	107.2 ± 24.4	108.7 ± 28.8	0.33
Laboratory evaluation			
pH	7.27 (7.13–7.44)	7.26 (7.08–7.35)	0.237
Base excess, mmol/L	− 9.7 (− 14.4 to − 3.2)	− 9.7 (− 18.3 to − 5.7)	0.353
Lactate, mg/dL	73.0 (36.0–91.0)	56.5 (36.0–100.0)	0.711
D-dimer, μg/mL	82.0 (27.6–117.7)	43.5 (15.9–99.9)	0.338
PT-INR	1.2 (1.0–1.4)	1.2 (1.0–1.4)	0.859
Trauma Score			
RTS	5.6 (2.6–6.6)	5.5 (3.5–6.6)	0.993
ISS	50.0 (41.0–66.0)	41.0 (29.0–50.0)	0.097
TRISS-Ps, %	43.3 (1.4–84.7)	36.9 (7.0–73.8)	0.76
Treatment outcome			
24-h mortality (%)	3 (20.0)	33 (35.9)	0.228
In-hospital mortality (%)	6 (40.0)	52 (56.5)	0.234

*REBOA* resuscitative endovascular balloon occlusion of the aorta, *GCS* Glasgow Coma Scale, *RR* respiratory rate, *SBP* systolic blood pressure, *BT* body temperature, *PT-INR* prothrombin time-international normalized ratio, *RTS* Revised Trauma Score, *ISS* Injury Severity Score, *TRISS-Ps* Probability of survival calculated by the Trauma and Injury Severity Score

The primary endpoint is presented in Table 2. Although the 24-h and in-hospital mortality were not significantly different between the two groups when compared using the *χ*^2^ test, REBOA was associated with a significant decrease in the in-hospital mortality when adjusted for age, RTS, ISS, pre-hemostasis-administrated RBCs, and the logarithm of time from admission to initiating surgery/IVR, using the multivariable logistic regression analysis. Adjusted odds ratio (OR) of survival was 7.430, and 95% CI was 1.081–51.062 (*p* = 0.041).

**Table 2:** Primary endpoint

Variable	Adjusted odds ratio of survival (95% CI)	*p*-value
REBOA	7.430 (1.081 - 51.062)	0.041

*REBOA* resuscitative endovascular balloon occlusion of the aorta, *CI* confidence interval

The secondary endpoints are presented in Tables 3 and 4. The amount of pre-hemostasis-administrated RBCs in the with REBOA group was higher than that in the without REBOA group when compared using the Mann–Whitney *U* test. However, there were no significant differences in any of the other parameters between both groups. Patients who were bleeding due to multiple injuries such as multiple facial bone fractures, skull base fractures with intracranial hemorrhage, mediastinal hematoma, chest wall hematoma, retroperitoneal hematoma, abdominal wall hematoma, or multiple extremities bone fractures, all of which were difficult to diagnose without CT, were not eligible for the hemostasis. These patients went into cardiac arrest before the hemostasis could be performed.

**Table 3:** Pre-hemostasis CT scan and hemostasis performance ratio and total amount of blood transfusions

	With REBOA (*n* = 15)	Without REBOA (*n* = 92)	*p*
Pre-hemostasis CT scan performance ratio (%)	12 (80.0)	60 (65.2)	0.258
The ratio of patients who underwent hemostasis (%)	14 (93.3)	81 (88.0)	0.643
Total amount of blood transfusions, units			
Red blood cells	16.0 (14.0–20.0)	16.0 (6.0–25.0)	0.175
Fresh frozen plasma	14.0 (6.0–20.0)	8.0 (4.0–18.0)	0.323

*CT* computed tomography, *REBOA* resuscitative endovascular balloon occlusion of the aorta

**Table 4:** Surgery/IVR-related characteristics and total amount of preoperative blood transfusions in the patients with surgery/IVR

	With REBOA (*n* = 14)	Without REBOA (*n* = 81)	*p*
Patients who underwent surgery for PH (%)	8 (57.1)	38 (46.9)	0.643
Time to initiation PH, min	53.0 (40.0–80.3)	57.0 (35.0–100.0)	0.908
Preoperative blood transfusions, mL			
Red blood cells	840.0 (560.0–1120.0)	560.0 (280.0–1120.0)	0.037
Fresh frozen plasma	120.0 (0.0–300.0)	0 (0–240.0)	0.104

*IVR* interventional radiology, *REBOA* resuscitative endovascular balloon occlusion of the aorta, *PH* primary hemostasis

The REBOA-related characteristics analyzed in this study are shown in Table 5. The time from admission to the start of balloon occlusion was 55.7 ± 34.2 min. From the REBOA group, 12 (80%) patients underwent CT before hemostasis. Among them, 6 (50%) patients underwent REBOA prior to CT. Hemostasis was initiated in 5 patients before they could receive REBOA. SBP just before the inflation of REBOA was 48.2 ± 10.5 mmHg. Total time for balloon occlusion was 32.5 ± 18.2 min. Two patients received both the intermittent and partial methods. A rest of 1–2 min was allowed after every 10 min of occlusion. Prior to removal, the volume of the inflated balloon was gradually reduced. The total occlusion time of the 2 patients were 50 and 60 min, respectively.

**Table 5:** REBOA related-characteristics

	With REBOA (*n* = 15)
Time from admission to start balloon occlusion, min	55.7 ± 34.2
The number of patients who underwent REBOA prior to CT (%)	6 (40.0)
The number of patients who started hemostasis before REBOA (%)	5 (33.3)
SBP just before inflation of the REBOA, mmHg	48.2 ± 10.5
Total length of balloon occlusion time, min	32.5 ± 18.2
The number of patients who received both intermittent and partial methods (%)	2 (13.3)
The morbidity of REBOA (%)	2 (13.3)
Arterial dissection (%)	2 (13.3)
Limb ischemia required below-the-knee amputation (%)	1 (6.7)
Acute kidney injury required HD (%)	1 (6.7)

*REBOA* resuscitative endovascular balloon occlusion of the aorta, *CT* computed tomography, *SBP* systolic blood pressure, *HD* hemodialysis

REBOA-related complications occurred in two patients without severe atherosclerotic changes. One middle-aged male patient, for whom the duration of balloon occlusion was 61 min, accompanied with arterial dissection of the right common iliac artery, left limb ischemia that required below-knee amputation, and acute kidney injury (AKI) that required hemodialysis. The time from admission to the initiation of laparotomy was 40 min without CT scan, and the time from admission to the inflation of the balloon (12-Fr REBOA set) was 50 min. The systolic blood pressure just before the balloon occlusion was 48 mmHg, the RTS was 6.085, and the ISS was 50. To prevent cardiac arrest, the balloon should not be deflated until the final stage of hemostasis. Another patient only required the dissection of the common iliac artery using a 12-French set. The dissections in both patients were conservatively treated, and their condition improved. AKI also improved with time. Eventually, both patients were able to resume a normal life.

## Discussion

The main finding of this study was that the use of REBOA was associated with reduced in-hospital mortality in patients with multiple severe torso trauma. Despite the use of REBOA in these patients, we were able to initiate surgery/IVR early in the with REBOA group than in the without REBOA group.

In this study, we used age, RTS, ISS, pre-hemostasis-administrated RBCs, and time from admission to the start of hemostasis as confounding variables. Age, RTS, and ISS were selected to exclude the influence of aging as well as physiological and anatomical differences [20, 21]. Furthermore, we added pre-hemostasis-administrated RBCs and time from admission to the start of hemostasis, which are the essentials for hemorrhage control in severely injured patients [22]. In addition, the results of time from admission to the start of hemostasis were skewed; therefore, we used the values for which logarithmic transformation was conducted. TRISS-Ps, lactate, base excess, and D-dimer values were used to evaluate the level of trauma severity between the two groups [23–25].

Inoue et al. [9] conducted a subgroup analysis of door-to-primary surgery time of < 60 min vs. ≥ 60 min and observed significant interactions, which may indicate that surgery time of ≥ 60 min could worsen in-hospital mortality. A delay in definitive hemostasis after REBOA may be one of the drawbacks that resulted in high mortality. Our analysis demonstrated that the time from admission to the start of surgery/IVR with REBOA was approximately 60 min, while the time from admission to deflating the balloon was approximately < 90 min. These results suggested that hemorrhage could be controlled within 90 min with surgery/IVR/REBOA in patients with multiple lethal torso trauma. Taken together, the most important factor required to successfully perform REBOA in patients with multiple severe trauma may be the rapid achievement of complete hemostasis. In other words, the use of REBOA should not be a cause of delay in hemostasis.

Our usage of REBOA had the following unique features: shorter occlusion time despite longer time from arrival to the start of balloon occlusion and lower SBP just before REBOA insertion compared with other instances in current literature [11, 13, 14]; the ratio of the patients who underwent REBOA was, low and resuscitative hemostasis had been initiated in five patients before the insertion of REBOA. We had used REBOA based on permissive hypotension [1–4]. Although cardiac arrest should be avoided, we considered the possible harmful effects of not just the long aortic occlusion time but also the unnecessary rising of central SBP due to early use of REBOA, especially in patients with multiple injuries. Hence, unnecessary use of REBOA should be avoided. REBOA might be used to increase afterload with redistribution of blood flow and prevent cardiac arrest, rather than perform hemorrhage control of the distal arteries. The result showed that REBOA might exert effects that can help preserve brain and coronary blood flow while improving outcomes. However, the optimal patient selection is difficult, and the other effects of REBOA remain unclear. Further investigations are needed regarding selection guidelines.

Another notable drawback is REBOA-related complications. Despite technological advancements, REBOA is associated with significant risks due to complications of vascular access and reperfusion ischemia [26, 27]. The serious complication of lower limb ischemia, which may lead to amputation, occurred with high frequency (3/24 patients) in a previous study [28]. In our study, lower limb ischemia, which also required amputation, occurred in 1 of 15 patients. Moreover, there were some REBOA-related complications. Although the exact reasons for the occurrence of the severe complications observed in this study were unclear, we believe that the large size and rigidity of the 12-Fr REBOA and the long occlusion time in patients whose arteries might be in a state of vasospasm could explain such complications. To save the patient’s life, some REBOA-related complications may even be inevitable, although all attempts should be made to avoid any adverse sequelae caused by REBOA-related complications. Some studies reported that small introducer sheaths for REBOA may be associated with fewer complications [27, 29] and that ultrasound should be optimized for REBOA [30]. Moreover, partial or intermittent REBOA should be considered in some cases [31, 32]. However, their effect remains unclear. Thus, prevention of REBOA-related morbidity is also important for successful trauma management with REBOA; however, we could not evaluate any possible harmful effects on mortality.

Statistically, this study may have some type 2 errors owing to a low-power analysis with the small sample size. However, our results suggest that there was non-inferiority at least with the REBOA group compared with the without-REBOA group. Therefore, we believe that these results may be of value in performing further prospective and multicenter studies.

There were several limitations to this study. Our study was conducted at a single center with a small sample size and retrospective study design. Our results were obtained using careful patient selection; however, the potential number of patients could have been higher. Therefore, there may be an obvious selection bias. Moreover, cases of severe trauma referred to our center were highly specific, complex, and had low interdisciplinarity. Medical equipment and techniques have progressed substantially. More cases should be assessed for investigation in future studies.

## Conclusions

The use of REBOA without a delay in initiating resuscitative hemostasis may improve the outcomes in patients with multiple injuries associated with severe trauma. Optimal usage of REBOA may be beneficial in preventing cardiac arrest and preserving brain and coronary blood flow without harmful effects. Further studies to assess optimal usage criteria are needed.

## Abbreviations

AKI: Acute kidney injury
AS: Angiography suite
CI: Confidence interval
CT: Computed tomography
ED: Emergency department
EPs: Emergency physicians
FFP: Fresh frozen plasma
ISS: Injury Severity Score
IVR: Interventional radiology
OR: Odds ratio
OR: Operating room
RBCs: Red blood cells
REBOA: Resuscitative endovascular balloon occlusion of the aorta
RTS: Revised trauma score
SBP: Systolic blood pressure
